# Impact of PINCH expression on survival in colorectal cancer patients

**DOI:** 10.1186/1471-2407-11-103

**Published:** 2011-03-22

**Authors:** Jasmine Lööf, Johan Rosell, Charlotte Bratthäll, Siv Doré, Hans Starkhammar, Hong Zhang, Xiao-Feng Sun

**Affiliations:** 1 Division of Tumour Biology, Systems Biology Research Centre, University of Skövde, Skövde, Sweden; 2 Department of Oncology, Linköping University Hospital, Linköping, Sweden; 3 Department of Pathology, Linköping University, Linköping, Sweden; 4 Department of Oncology, Linköping University, Linköping, Sweden

## Abstract

**Background:**

The adaptor protein PINCH is overexpressed in the stroma of several types of cancer, and is an independent prognostic marker in colorectal cancer. In this study we further investigate the relationship of PINCH and survival regarding the response to chemotherapy in colorectal cancer.

**Results:**

Paraffin-embedded tissue sections from 251 primary adenocarcinomas, 149 samples of adjacent normal mucosa, 57 samples of distant normal mucosa and 75 lymph node metastases were used for immunohistochemical staining. Stromal staining for PINCH increased from normal mucosa to primary tumour to metastasis. Strong staining in adjacent normal mucosa was related to worse survival independently of sex, age, tumour location, differentiation and stage (p = 0.044, HR, 1.60, 95% CI, 1.01-2.52). PINCH staining at the invasive margin tended to be related to survival (p = 0.051). In poorly differentiated tumours PINCH staining at the invasive margin was related to survival independently of sex, age and stage (p = 0.013, HR, 1.90, 95% CI, 1.14-3.16), while in better differentiated tumours it was not. In patients with weak staining, adjuvant chemotherapy was related to survival (p = 0.010, 0.013 and 0.013 in entire tumour area, invasive margin and inner tumour area, respectively), but not in patients with strong staining. However, in the multivariate analysis no such relationship was seen.

**Conclusions:**

PINCH staining in normal adjacent mucosa was related to survival. Further, PINCH staining at the tumour invasive margin was related to survival in poorly differentiated tumours but not in better differentiated tumours, indicating that the impact of PINCH on prognosis was dependent on differentiation status.

## Background

PINCH, particularly interesting new cystein-histidine-rich protein, was first identified in 1994 as an evolutionary conserved protein belonging to the LIM family, consisting of five LIM domains [1]. A LIM domain mediates protein interactions and consists of a protein-binding motif with a specific three-dimensional structure comprising a double zinc finger [2]. The PINCH gene is located to chromosome 2q12.2 and encodes a protein that functions as an adaptor protein [3]. PINCH is known to directly associate with two proteins: integrin-linked kinase (ILK) [4] and Nck-2 [5]. ILK is an intracellular serine/threonine protein kinase that is a constituent of integrin-mediated cell-matrix focal adhesions, structures that mediate cell adhesion and signal transduction between the extracellular matrix and the intracellular compartment [6]. Nck-2 is an adaptor protein capable of recognizing several key components of growth factor receptor kinase-signalling pathways [5]. PINCH binds Nck-2 and ILK by means of two separate LIM domains, LIM1 for ILK [4] and LIM4 for Nck-2 [5], and forms a multiprotein complex with these two proteins [4]. Thus, PINCH could provide a connection between the growth factor receptor and integrin-signalling pathways by mediating the interaction between Nck-2 and ILK.

The tumour-associated stroma is important in facilitating cancer growth and invasion, and PINCH expression has been shown to be up-regulated in tumour-associated stroma of several common cancer types, especially at the tumour invasive margin [7]. This indicates that PINCH could be involved in tumour progression. Further, PINCH functions in the integrin and growth factor signalling pathways, both important mediators of the tumour-stromal interaction. In concordance with the theory of PINCH promoting tumour progression, it has been shown that a high stromal expression of PINCH at the tumour invasive margin is related to worse prognosis in colorectal cancer [8].

In this study we further investigated the relationship of PINCH expression with survival and clinicopathological variables in colorectal cancer patients and found that PINCH expression at the tumour invasive margin or adjacent normal mucosa is independently related to prognosis. We also studied the effect of PINCH expression on outcome of adjuvant chemotherapy, and found that high PINCH expression could be related to treatment outcome. However, we did not find it to be an independent marker.

## Methods

### Patients

This study included 251 randomly selected patients with primary colorectal adenocarcinoma. The characteristics of the patients and tumours were obtained from surgical and pathological records at Linköping and Vrinnevi hospitals. The median age of the patients was 69 years (range 25-94 years). Tumour differentiation was graded as good, moderate, poor, or mucinous (including signet-ring cell carcinomas), and inflammatory infiltration was graded as weak, moderate or strong. Necrosis was graded as <10% and ≥ 10%. All patients underwent surgical resection at Linköping University Hospital (Linköping, Sweden) or Vrinnevi Hospital (Norrköping, Sweden), during the time period of 1973 to 2001. After surgery the patients were considered to have adjuvant chemotherapy, which was given to 27 patients. The main indication for adjuvant treatment was radically resected stage II or III tumours with additional risk factors (i.e. vascular invasion and poor differentiation) in colon cancer. Also, one rectal cancer patient with a stage III tumour and additional risk factors was included. Depending on various study protocols active at each time, the drugs and administration schedule differed (Table 1). The study was approved by the local human research ethics committee.

**Table 1:** Status of adjuvant therapy in colorectal cancer patients

Adjuvant therapy	N
5-Fluorouracil (5-FU)+ leukovorin (Nord)	8
Panorex	6
Xeloda	5
5-FU + leukovorin (Mayo)	5
Fluorouracil + levamisole	1
Panorex + 5-FU + leukovorin (Mayo)	1
Other chemotherapy	1
Radiotherapy	1
Unknown	1

Nord and Mayo: administration schedules. Nord: drugs are administered as injections on day 1 and 2. Mayo: drugs are administered as injections on day 1-5.

### Immunohistochemistry

For immunohistochemical staining 5 μm formalin-fixed, paraffin-embedded tissue sections were used. The study included 251 samples of primary tumour, among them, 149 had adjacent normal mucosa. There were also 57 samples of distant normal mucosa taken from the margin of the distant resection and 75 samples of regional lymph node metastasis. The sections were incubated at 60 oC overnight, then deparaffinized in xylene and rehydrated in a series of ethanol with decreasing concentrations. In order to expose masked epitopes the sections were boiled in 0.01 M Tris-EDTA buffer (pH 9.0) in a high pressure cooker at 125 oC for 30 seconds after which the sections were cooled to 90 oC for 10 seconds and then kept in room temperature for 10 minutes. After washing in phosphate buffered saline (PBS, pH 7.4) the sections were incubated in a 2% H2O2- methanol solution for 20 minutes in order to block endogenous peroxidase activity. The sections were then washed in PBS and incubated with protein block solution (Dako, Carpinteria, CA) for 10 minutes. Subsequently, the sections were incubated with the primary antibody, a polyclonal rabbit anti-PINCH (Rockland Laboratories, Gilbertsville, PA) (2 μg/ml), for 1 hour. After washing in PBS, the sections were incubated with the secondary antibody (Dako ChemMate EnVision Detection Kit; Dako, Glostrup, Denmark), a goat anti- rabbit/mouse polymeric HRP conjugate for 25 minutes, followed by washing with PBS. The peroxidase reaction was performed using 3,3'- diaminobenzidine chromogen (Dako) and finally the sections were counterstained with haematoxylin. Sections known to stain positively were used as positive controls. For the negative control, PBS was used instead of the primary antibody.

The sections were microscopically examined and scored independently by two investigators without any knowledge of the clinicopathological data. The staining intensity was determined for distant and adjacent normal mucosa, the entire tumour area, the inner tumour area, and the invasive margin of the tumours, and was scored as negative, weak, moderate or strong. Because of the clinicopathological similarities we combined the negatively, weakly and moderately stained groups as one group (weak group), and compared it to the strong group in the statistical analysis. The cases with discrepant scoring were re-evaluated individually until both investigators agreed on the scoring. The remaining were re-examined by the two investigators together using a dual-headed microscope in order to reach a consensus score. To avoid artificial effects, areas with necrosis, poor morphology, or in the margins of the sections, were not considered.

### Statistical analysis

The statistical analyses were performed using SPSS version 17.0. The χ2 test was used to determine differences in PINCH expression among normal mucosa, primary and metastatic colorectal cancer, as well as relationship of PINCH expression in primary colorectal cancer with clinicopathological variables. Cox's proportional hazard model was used to test relationship between PINCH expression and patients' survival. The Kaplan- Meier method was used to calculate survival curves. Two-sided p-values of <0.05 were considered statistically significant.

## Results

### PINCH expression in normal mucosa, primary tumour and metastasis

**Figure 1:**
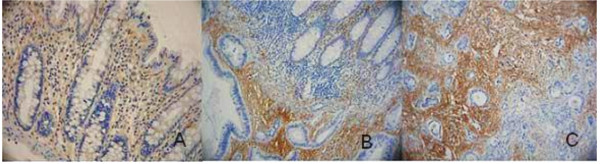
**Immunohistochemical staining for PINCH**. **A) **Weak PINCH immunohistochemical staining (yellow-brown colour) in distant normal mucosa. **B) **Moderate staining in the adjacent normal mucosa (top right corner of the picture) and strong staining in the primary tumour (lower left of the picture). **C) **Even stronger staining in lymph node metastasis.

**Figure 2:**
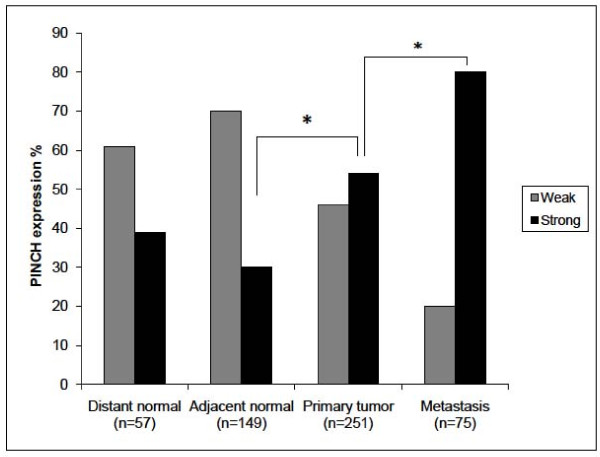
**PINCH expression in distant and adjacent normal mucosa, primary tumour and lymph node metastasis**. The frequency of the strong PINCH expression is significantly increased from adjacent normal mucosa to primary tumour (p = 0.0001) and from primary tumour to metastasis (p = 0.007).

PINCH staining was present in the cytoplasm of the fibroblasts in the stroma, whereas normal epithelial and tumour cells, except for two cases, did not show any staining (Figure 1). Of the 57 distant normal mucosa samples, 61% were weakly stained and 39% were strongly stained. As for the 149 adjacent normal mucosa samples, 71% were weakly stained, and 29% of the samples were strongly stained. Of the 251 primary tumour samples 46% had weak staining and 54% strong staining, and of 75 metastases, 20% were weakly stained, and 80% strongly stained. The frequency of the strong PINCH staining was shown to be significantly increased from adjacent normal mucosa to primary tumour (p = 0.0001), and from primary tumour to metastasis (p = 0.007) (Figure 2). The intensity of PINCH staining did not significantly differ between distant normal and adjacent normal mucosa (p = 0.077), nor between distant normal mucosa and primary tumour (p = 0.16).

### PINCH expression in relation to patient survival and other clinicopathological variables

**Figure 3:**
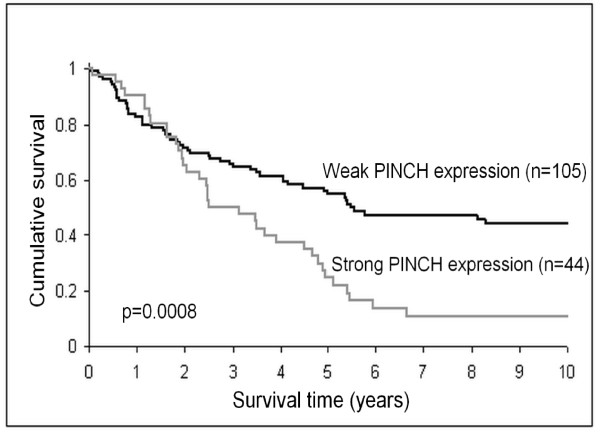
**PINCH expression in adjacent normal mucosa in relation to patient survival**. Strong expression of PINCH in adjacent normal mucosa is significantly related to worse survival (p = 0.0008).

**Figure 4:**
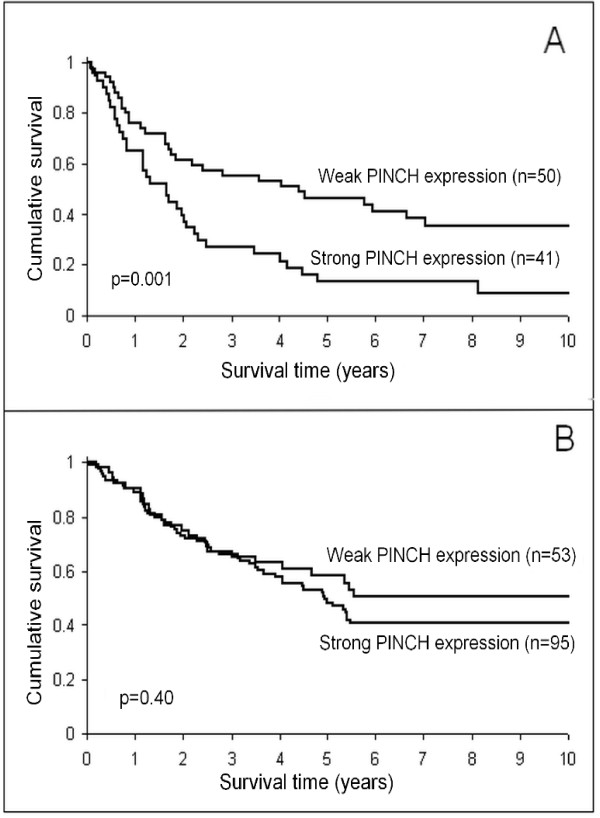
**PINCH expression at the primary tumour invasive margin in relation to patient survival**. **A) **Poorly differentiated tumours. **B) **Better differentiated tumours. Strong expression of PINCH is related to worse survival in poorly differentiated tumours (p = 0.001) but not in better differentiated tumours (p = 0.40).

In adjacent normal mucosa, strong stromal staining for PINCH was related to poorer survival (p = 0.0008, Figure 3). In the multivariate analysis the relationship was still significant independently of sex, age, tumour location, differentiation and stage (p = 0.044, HR, 1.60, 95% CI, 1.01-2.52, Table 2). Strong PINCH staining at the primary tumour invasive margin also tended, although not significantly, to be related to worse survival (p = 0.051). Furthermore, in poorly differentiated tumours (including mucinous and signet-ring cell carcinomas), the strong staining at the invasive margin was significantly related to worse survival both in the univarite analysis (p = 0.001, Figure 4A), and in the multivariate analysis including sex, age and stage (p = 0.013, HR, 1.90, 95% CI, 1.14-3.16, Table 3). However, in better differentiated tumours (including well and moderately differentiated tumours) PINCH staining was not related to survival (p = 0.40, Figure 4B). Strong PINCH staining at the tumour invasive margin was also related to less inflammatory infiltration (p = 0.047, Table 4) and better differentiation (p = 0.005, Table 4).

**Table 2:** Cox multivariate regression analysis of PINCH expression in adjacent normal mucosa, sex, age, tumour location, grade of differentiation and stage in relation to patient survival in colorectal cancer

Variable	N (%)	Death (%)	HR	95% CI	*P*-value
**PINCH expression**					
Weak	102 (70)	52 (51)	1.00	-	-
Strong	44 (30)	35 (80)	1.60	1.01-2.53	0.044
**Sex**					
Male	83 (57)	52 (63)	1.00	-	-
Female	63 (43)	35 (56)	0.82	0.53-1.28	0.39
**Age (years)**					
≤ 69	70 (48)	46 (66)	1.00	-	-
> 69	76 (52)	41 (53)	1.51	0.96-2.39	0.075
**Tumour Location**					
Colon	77 (53)	46 (60)	1.00	-	-
Rectum	69 (47)	41 (59)	1.16	0.75-1.79	0.51
**Differentiation**					
Well+moderate+poor	121 (83)	71 (59)	1.00	-	-
Mucinous	25 (17)	16 (64)	0.95	0.55-1.65	0.85
**Stage**					
I	17 (12)	3 (18)	1.00	-	-
II	34 (23)	15 (44)	3.32	0.95-11.62	0.061
III	64 (44)	40 (63)	6.98	2.11-23.06	0.0014
IV	31 (21)	29 (94)	28.71	8.24-100.03	< 0.0001

HR: Hazard ratio; 95% CI: 95% confidence interval. (69 years = median age at operation)

**Table 3:** Cox multivariate regression analysis of PINCH expression at tumour invasive margin, sex, age and stage in poorly differentiated tumours in relation to patient survival in colorectal cancer

Variable	N (%)	Death (%)	HR	95% CI	*P*-value
**PINCH expression**					
Weak	50 (55)	30 (60)	1.00	-	-
Strong	41 (45)	36 (88)	1.90	1.14-3.16	0.013
**Sex**					
Male	48 (53)	34 (71)	1.00	-	-
Female	43 (47)	32 (74)	1.10	0.67-1.81	0.69
**Age (years)**					
≤ 69	51 (56)	40 (78)	1.00	-	-
> 69	40 (44)	26 (65)	1.37	0.81-2.32	0.24
**Stage**					
I+II	18 (20)	7 (39)	1.00	-	-
III	38 (42)	26 (68)	2.75	1.16-6.56	0.022
IV	35 (38)	33 (94)	9.09	3.63-22.75	< 0.0001

HR: Hazard ratio; 95% CI: 95% confidence interval. (69 years = median age at operation)

**Table 4:** Intensity of PINCH expression at the primary tumour invasive margin in relation to clinicopathological variables

Variable PINCH expression at tumour invasive margin			
	**Weak (%)**	**Strong (%)**	***P-*value**
	
**Sex**			0.97
Male	55 (53)	74 (54)	
Female	48 (47)	64 (46)	
**Age (years)**			0.97
≤ 69	52 (50)	70 (51)	
> 69	51 (50)	68 (49)	
**Tumour location**			0.28
Colon	63 (62)	75 (55)	
Rectum	39 (38)	62 (45)	
**Stage**			0.34
I	13 (13)	9 (7)	
II	22 (21)	28 (21)	
III	44 (43)	56 (42)	
IV	24 (23)	41 (31)	
**Differentiation**			0.005
Better	53 (52)	95 (69)	
Poorer	50 (48)	42 (30)	
**Inflammatory infiltration**			0.047
Weak	25 (26)	54 (40)	
Moderate	43 (44)	52 (39)	
Strong	30 (31)	28 (21)	
**Necrosis**			0.32
< 10%	58 (59)	70 (53)	
≥ 10%	40 (41)	63 (47)	

### PINCH expression in relation to chemotherapy

In the univariate analysis, patients with weak stromal staining for PINCH in the entire primary tumour (p = 0.010), inner tumour area (p = 0.013) or at the invasive margin (p = 0.013) receiving adjuvant chemotherapy had significantly better survival than those without chemotherapy. In patients with strong staining for PINCH in the entire tumour (p = 0.13), inner tumour area (p = 0.16) or at the invasive margin (p = 0.16) chemotherapy was not related to survival. However, in the multivariate analysis there was no significant relationship between PINCH staining and chemotherapy outcome (data unshown).

## Discussion

Predictive and prognostic markers in colorectal cancer patients have been the subject of intense research. The determination of prognosis predominantly relies on the histopathological examination, although there are certainly other factors influencing survival. Approaches are being made to improve prognostic methods, including analyzing additional histopathological factors and molecular and genetical markers. Although these markers are promising they are not yet routinely used. Potential markers include, for example, allelic imbalances, chromosomal instability, expression of oncogenes, loss of tumour suppressor genes, markers of proliferation, angiogenesis, inflammation and cell adhesion as well as genes involved in the response to chemo- and radiotherapy.

In this study we have shown that the intensity of stromal immunohistochemical staining for PINCH was increased from normal mucosa to primary tumour and from primary tumour to lymph node metastasis in colorectal cancer. We have also shown that strong staining for PINCH in normal adjacent mucosa was related to worse survival. Furthermore, in poorly differentiated tumours, PINCH staining at the tumour invasive margin was significantly related to survival, while in better differentiated tumours it was not. The pattern of PINCH staining increasing from normal tissue to tumour and from tumour to metastasis has been seen in previous studies [8] and indicates that PINCH is involved in tumour progression and invasion. PINCH up-regulation in the stroma of oral squamos cell carcinoma also predicts lymph node metastasis, further implicating PINCH in invasion and metastasis [9]. It has previously been shown that PINCH staining at the tumour invasive margin is an independent prognostic marker in colorectal cancer [8], which we have also seen a tendency towards in the current study. This further supports the idea that PINCH, through involvement in the tumour-stromal interaction, promotes tumour invasiveness. The finding that the intensity of PINCH staining in poorly differentiated tumours was significantly related to survival while in better differentiated tumours it was not suggests that the effect of PINCH expression on the aggressiveness of the tumour could be dependent on differentiation status. It seems as the impact of PINCH expression on survival is limited in well differentiated tumours. As it has previously been seen [8], we also found strong PINCH expression to be related to better differentiation. Poor differentiation is correlated to worse survival [10], nevertheless strong expression of PINCH at the tumour invasive margin seems to be related to a worse prognosis. Further, PINCH staining in the adjacent normal mucosa was found to be related to survival, which has not been reported previously. Although histologically the mucosa adjacent to a tumour appears normal, it is from colorectal cancer patients and may differ biologically from the normal mucosa of a healthy individual. Because of the proximity of the adjacent normal mucosa with the invasive margin of the tumour, it is possible that this seemingly normal tissue is already involved in signalling and interactions with the tumour. The finding that PINCH expression in the adjacent normal mucosa is related to survival implicates PINCH in the biological changes occurring in the mucosa around a tumour.

The interaction between tumour and stroma has been recognized as an important factor influencing tumour growth and progression [11,12]. PINCH is involved in several signalling pathways important to the tumour-stromal interaction by functioning as an adaptor protein in the integrin- and growth factor signalling pathways [5,13]. Loss of PINCH in *c. elegans *results in a phenotype identical to integrin null mutants [3], indicating that PINCH is required for integrin signalling, one of the key components in the cell-stromal interaction. Integrin-mediated adhesion to the extracellular matrix results in the stimulation of various intracellular pathways important in the regulation of cell attachment, migration, proliferation [14,15], survival and apoptosis [16]. PINCH directly associates with the proteins integrin-linked kinase (ILK) and Nck-2, and assembly of the multi-protein complex containing PINCH, ILK and Nck-2 prevents the proteolytic degradation of the proteins that are part of the complex [13]. Therefore, an increase in PINCH expression will increase the stability of the other proteins in the complex, thus increasing ILK and Nck-2 signalling. Possibly, the aggressive behaviour of tumours with a high stromal expression of PINCH can be explained by an up-regulation of the signalling pathways that are dependent on the adaptor function of PINCH. It is not clear which factors during tumour progression that influence the expression of PINCH itself. Studies of epithelial-to-mesenchymal transition (EMT) in renal tubuli have shown PINCH mRNA expression to be increased in response to transforming growth factor (TGF)-β1 [17], a known inducer of EMT in several biological systems, including renal tubuli [18]. TGF-β1 has been implicated in the progression of colorectal cancer [19]; therefore it is possible that TGF-β1 could be involved in the regulation of PINCH in colorectal tumours.

ILK is a serine/threonine protein kinase that associates with the cytoplasmic domain of the integrin β1 and β3 subunits, thereby regulating integrin mediated signal transduction [6]. PINCH and ILK have been shown to be indispensable for proper control of cell shape change, motility and survival [13]. ILK is activated through the PI3K-signalling pathway and functions in cell survival by regulating the PKB/Akt signalling pathway [20]. Up-regulation of ILK in tumour cells is observed in several types of cancer, and is associated with tumour stage, metastasis and worse prognosis [21-23]. ILK overexpression promotes an invasive phenotype, induces in vivo tumourigenesis [24], anchorage independent cell growth [6] and anchorage independent cell cycle progression [25]. Clearly, ILK plays an oncogenic role when overexpressed in epithelial cells. However, in our study we found PINCH to be overexpressed in the stroma, therefore ILK signalling may be increased in stromal cells. The question is therefore how increased ILK signalling in stromal cells can influence the aggressiveness of the tumour.

The fibroblasts of the tumour associated stroma can affect tumour development by secreting soluble factors such as vascular endothelial growth factor (VEGF) [26] and matrix metalloproteinases (MMP:s) [27]. ILK contributes to tumour progression by increasing the expression of VEGF via the activation of PKB/Akt and HIF-1α, thereby stimulating angiogenesis [28]. Angiogenesis is a prerequisite for tumour growth and progression [29], and the levels of angiogenetic factors such as VEGF are related to prognosis in several types of cancer, including colorectal cancer [30]. Angiogenesis is speculated to contribute to metastasis by increasing the number of leaky vessels into which the tumour cells can intravasate [31] and the VEGF expression has been shown to be related to tumour stage [32], lymph node metastasis [33,34], distant metastasis [34] and depth of tumour invasion [32]. Furthermore, at our laboratory, a recent study (unpublished data) of rectal cancer patients showed that PINCH expression was related to blood- and lymph vessel density, implicating PINCH as a regulator of angiogenesis.

Further contributing to an invasive phenotype, ILK overexpression stimulates the expression of matrix metalloproteinase -9 (MMP-9) [35]. Metalloproteinases are zinc-dependent endopeptidases that degrade components of the extracellular matrix [36], a process that is necessary for angiogenesis, tumour invasion and metastasis to occur [37]. Increased expression of MMP:s is associated with tumour invasion [38,39] and metastasis [40,41]. In particular, MMP-9 plays a key role in the degradation of several components of the ECM, including type IV, V and XI collagen, gelatin and laminin [42], and has been found to be over-expressed in several types of cancer and to be associated with a worse prognosis [43,44].

Remodelling of the extracellular matrix is a necessary process in order for a tumour to grow and progress [45]. The remodelling consists not only of the breakdown of ECM, but also the neosynthesis of certain ECM components such as fibronectin [46]. Assembly of fibronectin is regulated by ILK in a process requiring PINCH [24]. Therefore, an increase in PINCH expression at the tumour invasive margin could be associated with enhanced assembly of fibronectin matrix. Since the fibronectin matrix has a major impact on cell adhesion, migration, and cell growth [47,48], an increased assembly could stimulate the migratory and invasive capacity of the tumour cells. A high expression of fibronectin in tumour stroma has been found to be correlated to lymph node metastasis, proliferation and worse survival [49]. The interaction between fibronectin and tumour cells activates various signalling pathways involved in tumour progression, leading for example to the increased expression of metalloproteinases MMP-2 and -9 [50,51].

ILK is connected to the growth factor signalling pathways through PINCH, since PINCH forms a multiprotein complex with ILK and the adaptor protein Nck-2 [5]. Nck-2 recognizes several key components of growth factor receptor kinase-signalling pathways, including EGF receptors, PDGF receptor-β and insulin receptor substrate-1(IRS-1) [5]. Thus, PINCH provides a physical connection between the growth factor receptor-signalling pathways and the integrin-mediated pathways by connecting ILK and the integrins with EGF-and PDGF receptors and IRS-1 [4]. Through the complex formation with Nck-2, increased PINCH expression may be associated with an up-regulation of the growth factor signalling pathways. Growth factors are important regulators of the tumour-stromal interaction, and for some carcinomas an increase in growth factor receptors in stromal cells is thought to be an essential part in tumour to stroma signalling and hence tumour growth and progression [52,53].

Since PINCH expression is related to tumour progression and prognosis, we were interested in investigating whether PINCH was also related to response to treatment. In our group of patients, 27 patients received adjuvant chemotherapy. In the univariate analysis we found chemotherapy to be significantly related to survival in patients with weak stromal staining for PINCH at the invasive margin, while in patients with strong staining there was no relationship between chemotherapy and survival. This indicates that PINCH could be one factor influencing the outcome of adjuvant chemotherapy. However, in the multivariate analysis we found no significant correlation between chemotherapy outcome and PINCH expression. Possibly, this could be due to the relationship of PINCH expression with inflammatory infiltration and differentiation grade, known prognostic factors in colorectal cancer [10,54]. Further, the relatively low number of patients may contribute to the lack of significant results. More results would require a prospective setting; as with many studies of prognostic or predictive markers the major weakness of this study is the use of retrospective, non-randomized material as well as the low number of the patients.

## Conclusions

Stromal immunohistochemical staining for PINCH in normal mucosa adjacent to a tumour was found to be related to survival in colorectal cancer patients. Although appearing histologically normal, biological changes may occur in the mucosa near a tumour, possibly affecting the behaviour of the tumour. Further, PINCH staining at the tumour invasive margin was related to survival in poorly differentiated tumours but not in better differentiated tumours, indicating that the impact of PINCH on prognosis is dependent on differentiation status. Regarding treatment, adjuvant chemotherapy was significantly related to survival in patients with weak stromal staining for PINCH, but not in patients with strong staining. Thus, patients with weak PINCH staining seem to benefit more from adjuvant chemotherapy than patients with strong staining. However, these results could not be confirmed in a multivariate analysis. Taken together, our results indicate that PINCH could be one factor influencing the prognosis in colorectal cancer patients.

## Authors' contributions

JL carried out the experiments and drafted the manuscript. JR performed the statistical analysis. CB and HS collected clinical data and raised clinical questions. SD collected pathological material and data, HZ confirmed pathological variables by reading slides. XFS conceived of the study, participated in the design and helped in the drafting of the manuscript. All authors read and approved the final manuscript.

## Pre-publication history

The pre-publication history for this paper can be accessed here:

http://www.biomedcentral.com/1471-2407/11/103/prepub
